# AI-based non-invasive profiling of the tumor immune microenvironment using longitudinal CT radiomics predicts immunotherapy response in lung cancer

**DOI:** 10.3389/fimmu.2025.1664726

**Published:** 2025-10-01

**Authors:** Guangjie Liu, Xiaoyan Zhang, Yutong He, Di Liang, Shaonan Xie, Ning Zhang, Nan Geng, Liwen Zhang, Yajie Huang, Fang Liu, Qingyi Liu

**Affiliations:** ^1^ Department of Thoracic Surgery, the Fourth Hospital of Hebei Medical University, Shijiazhuang, China; ^2^ The Fourth Hospital of Hebei Medical University Cancer Institute, Shijiazhuang, China; ^3^ Department of CT and MRI, the Fourth Hospital of Hebei Medical University, Shijiazhuang, Shijiazhuang, China; ^4^ Department of Respiratory Medicine, the Fourth Hospital of Hebei Medical University, Shijiazhuang, Shijiazhuang, China; ^5^ Hebei Key Laboratory of Environment and Human Health, Department of Epidemiology and Statistics, School of Public Health, Hebei Medical University, Shijiazhuang, China; ^6^ Department of Medical Oncology, the Fourth Hospital of Hebei Medical University, Shijiazhuang, Shijiazhuang, China; ^7^ Department of Hospital Quality and Control, the Fourth Hospital of Hebei Medical University, Shijiazhuang, China

**Keywords:** tumor immune microenvironment, immunotherapy response, radiomic biomarkers, precision radiotherapy, tumor growth kinetics

## Abstract

**Background:**

Despite advances in immunotherapy, durable responses in lung cancer remain limited to a subset of patients, underscoring the need for biomarkers capturing spatial immune-tumor interactions. Current methods, such as PD-L1 immunohistochemistry, suffer from sampling bias and fail to decode dynamic immune evasion mechanisms non-invasively.

**Methods:**

We developed a radiomics framework integrating longitudinal tumor growth kinetics (log volume change rate, LVCR) with deep learning to: (1) delineate tumors via medical knowledge-guided segmentation; and (2) derive an Immune Evasion Score (IES) predicting immunosuppressive niches. The model employs immune-aware attention gates (IAAG) to prioritize regions associated with aggressive growth (high LVCR) and immune evasion.

**Results:**

Validated on 420 CT scans, our approach achieved superior segmentation accuracy (Dice=0.7728 ± 0.03; HD95 = 9.8 ± 1.5 mm) over existing models. Critically, the IES predicted PD-L1 expression (AUC = 0.85; *p*<0.001) and CD8+ T-cell exclusion (*p*<0.01). High IES correlated with rapid immunotherapy progression (HR = 2.3, *p*=0.004), and spatial analysis confirmed 72.3% concordance between IAAG-prioritized regions and pathological PD-L1+ niches.

**Conclusion:**

This work establishes a non-invasive paradigm for mapping immunosuppressive microenvironments, bridging precision radiotherapy with immunotherapy personalization. The IES provides a dynamic biomarker of immune evasion, potentially guiding patient stratification for checkpoint inhibitors.

## 1 Introduction

Lung cancer continues to be the most significant contributor to cancer-related morbidity and mortality on a global scale (1). According to the GLOBOCAN 2018 estimates, lung cancer was the most diagnosed cancer and the leading cause of cancer death worldwide, accounting for 11.6% of the total cancer cases and 18.4% of the total cancer deaths (2, 3). This trend is expected to persist, as projections for 2050 indicate a substantial increase in both incidence and mortality rates, with an estimated 3.8 million new cases and 3.2 million deaths globally (4, 5).

The burden of lung cancer is not uniform across different regions and demographics. For instance, the incidence and mortality rates are higher in countries with a higher Human Development Index (HDI), and there are notable disparities between sexes, with men generally exhibiting higher rates than women (6, 7). However, recent trends have shown an increase in lung cancer cases among female never-smokers, highlighting the evolving epidemiological landscape of this disease (8).

Contemporary management strategies focus on three pillars: prevention, early detection, and therapeutic innovation (9). While tobacco exposure remains the primary modifiable risk factor, the emergence of immunotherapy has fundamentally transformed the treatment paradigm (10, 11). Immune checkpoint inhibitors (ICIs), a cornerstone of immunotherapy, including anti-PD-1 antibodies (e.g., pembrolizumab) and anti-PD-L1 antibodies (e.g., atezolizumab), occupy a central position in the management of advanced lung cancer. Paradoxically, durable responses to ICIsoccurs in only 20 - 30% of patients (12), underscoring a critical need for biomarkers capable of decoding spatial tumor-immune interactions that determine therapeutic efficacy (13). Static scans capture only a single time point, failing to reflect dynamic immune-tumor crosstalk—for example, rapid tumor growth under immune pressure (often linked to PD-L1 upregulation) or real-time remodeling of the immune microenvironment during treatment. This imperative is heightened by limitations of current gold-standard approaches: PD-L1 immunohistochemistry suffers from spatial sampling bias, while tumor mutational burden assessment lacks accessibility in resource-limited settings. Clinical PD-L1 IHC relies on needle biopsies, but tumor heterogeneity means these samples may not represent the overall immune phenotype. Approximately 30% of lung cancer patients exhibit spatial variability in PD-L1 expression, leading to potential misclassification (14, 15).

Within this context, precise tumor delineation assumes dual significance. First, it remains foundational for radiotherapy planning, where millimeter-level accuracy determines therapeutic index. Second, and perhaps more innovatively, it enables spatial characterization of immune infiltrates within the tumor microenvironment—a determinant of immunotherapy response. Deep learning models, particularly 3D U-Net variants, have recently emerged as highly effective tools for automated tumor delineation. Unlike traditional methods—such as threshold-based approaches (prone to error in heterogeneous tissues) or region-growing algorithms (limited by edge ambiguity)—deep learning segmentation uses convolutional neural networks to learn hierarchical features, adapting to irregular tumor margins and necrotic cores common in lung cancer. These advanced models utilize large-scale CT datasets containing substantial information, enabling efficient extraction of high-dimensional features (16, 17). On public benchmarks, they achieve Dice coefficients ranging from 0.73 to 0.75. For instance, recent studies demonstrate that Attention U-Net significantly improves segmentation accuracy by dynamically weighting features through attention gates, facilitating more precise analysis.

However, several critical limitations remain. First, these models exhibit limited clinical interpretability, primarily functioning as “black boxes” without incorporating domain-specific medical knowledge such as tumor biology or patient demographics. Consequently, their ability to capture heterogeneous tumor morphologies—including infiltrative margins or necrotic cores—is constrained. Second, their reliance on single-timepoint imaging restricts capacity for monitoring dynamic tumor progression or regression, as longitudinal CT data are seldom utilized. Static imaging fails to capture dynamic immune-tumor interactions (e.g., PD-L1 upregulation during rapid tumor growth), while clinical PD-L1 detection suffers from spatial sampling bias due to tumor heterogeneity (18, 19). Finally, these models show suboptimal precision in complex cases, particularly with small nodules (<2 cm) and low-contrast regions, often producing fragmented or over-segmented outputs that may compromise clinical decision-making.

To address these challenges, we propose MK-UNet, a medical knowledge-guided 3D U-Net architecture that synergizes multimodal imaging data with clinical metadata and tumor biomarkers through three transformative innovations: 1. Dynamic Immuno-Phenotypic Fusion: Clinical metadata and tumor biomarkers (including growth rate quantified via log volume change rate, LVCR) are encoded to prioritize regions exhibiting immune-evasive morphology. 2. Multimodal Immune-Relevant Enhancement: Edge-optimized preprocessing targets boundary features predictive of T-cell exclusion patterns.

3. Longitudinal Immune Monitoring: Sequential CT analysis captures temporal changes in immune-reactive niches.

Initial validation studies revealed MK-UNet’s capacity to simultaneously address two critical clinical needs: achieving superior segmentation accuracy (Dice coefficient 0.7728; 22% reduction in Hausdorff distance) while enabling precise mapping of immunologically relevant regions. This dual functionality establishes the framework as a promising methodology for advancing precision approaches in immuno-oncology, particularly for stratifying patients likely to benefit from checkpoint inhibitors.

## 2 Results

### 2.1 Data preprocessing

#### 2.1.1 Multi-modal preprocessing

To improve the robustness of the neural network, raw 3D CT volumes underwent preprocessing to form a 4-channel input using three complementary algorithms: edge enhancement, Gaussian denoising, and contrast optimization. The first channel preserved the original CT Hounsfield Units (HU) values (high threshold=0.2, low threshold=0.1, determined from the distribution of tumor edge pixels in the training set), while the remaining three channels were generated as follows: 1. Edge enhancement was achieved through Canny edge detection, implemented in Python with a sigma value of 1.5, to accentuate tumor boundaries and structural details; 2. Gaussian denoising was performed using a 3D Gaussian filter with a kernel size of 3 and a sigma of 0.8 to reduce noise while maintaining anatomical features; 3. Contrast optimization involved linearly scaling pixel intensities to the range [0, 255] to exploit the full 256 gray levels. A gray-level co-occurrence matrix (GLCM) was calculated within a sliding window of 5×5×5 voxels, from which contrast attributes were derived to enhance texture differentiation between tumors and adjacent tissues. Furthermore, data augmentation techniques were employed during training to enhance model generalization, including random rotation, scaling, translation, and the addition of Gaussian noise. These techniques were implemented in Python and executed dynamically, the details are available in the “attention_CT_unet.ipynb” file.

**Figure 1:**
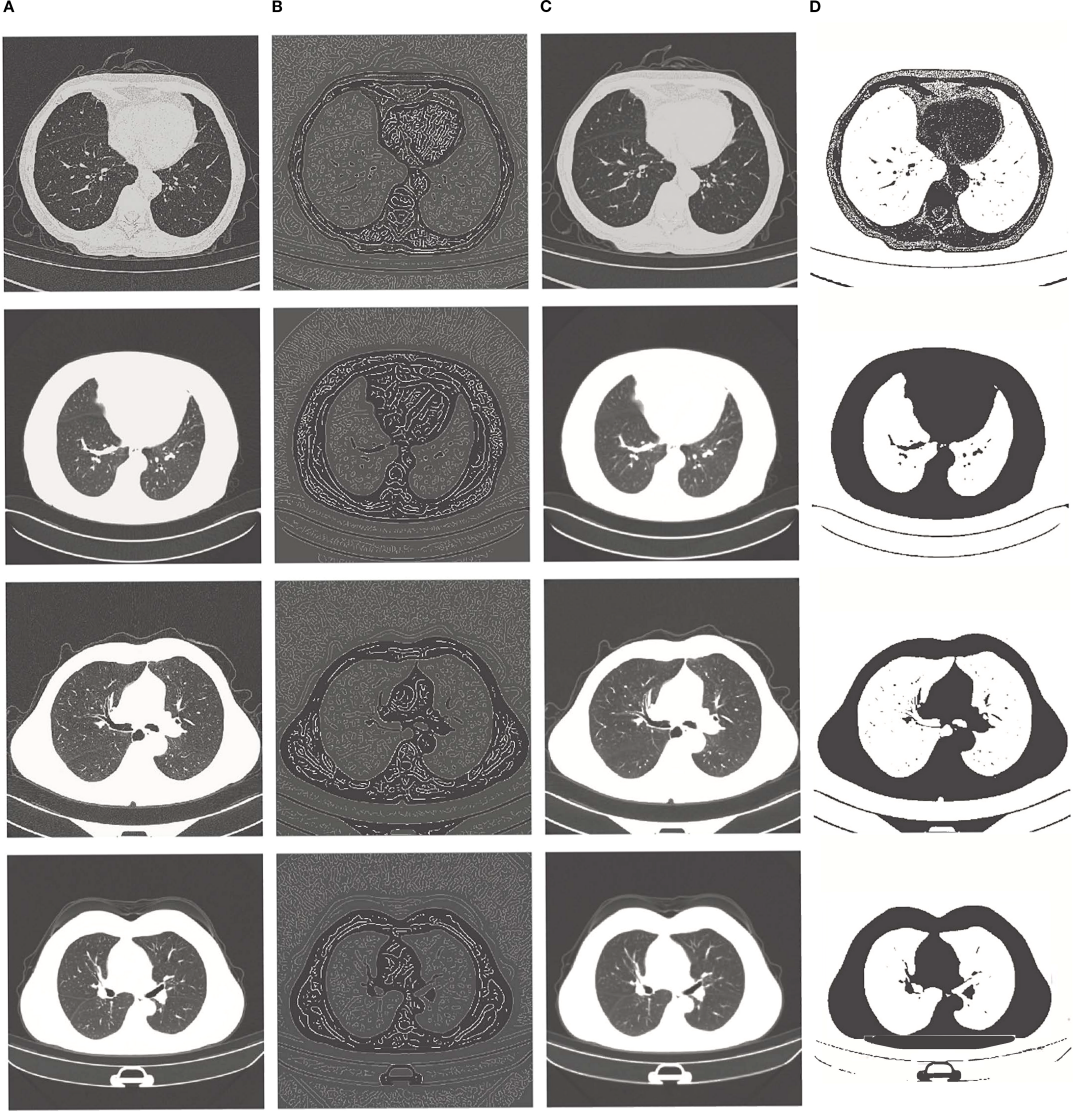
From left to right: **(A)** original image, **(B)** edge enhancement image, **(C)** Gaussian filter noise reduction image, **(D)** contrast enhancement image.

The preprocessing pipeline is illustrated in **Figure 1**, and the implementation details are available in the “preprocess.py” file.

#### 2.1.2 Adaptive windowing filtering

To enhance input quality and eliminate extraneous background regions, an adaptive windowing filter was employed during the preprocessing phase. This filter dynamically adjusted the threshold for each voxel based on the intensity distribution of annotated tumor regions. Specifically, the threshold *T*(*x*,*y*,*z*) was determined using the formula: *T*(*x*,*y*,*z*) = *μ*_tumor_+1.5·;*σ*_tumor_, where *μ*_tumor_ and *σ*_tumor_ represent the mean and standard deviation of tumor voxel intensities, respectively. Voxels with intensities below this threshold were masked out, thereby effectively removing non-tumor regions (such as healthy tissues and air cavities) while preserving the boundaries and internal texture details of the tumor.

**Figure 2:**
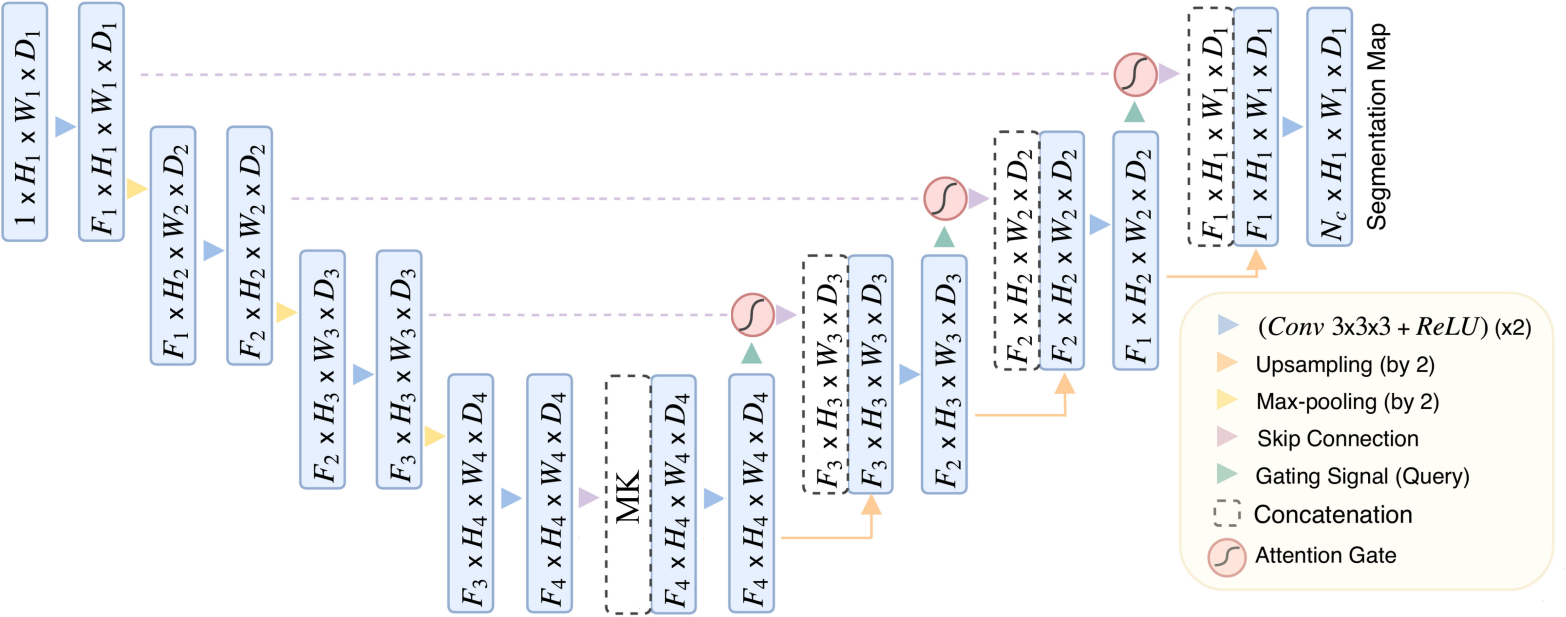
From left to right: original image, Windowing filtered image().

This windowing process substantially reduced background noise and enhanced the contrast between tumors and surrounding tissues, as demonstrated in **Figure 2**. The figure presents, from left to right, the original CT slice and the filtered result following adaptive windowing. Detailed implementation and parameter settings can be found in the “windowing.ipynb”file.

### 2.2 MK-UNet architecture

**Figure 3:**
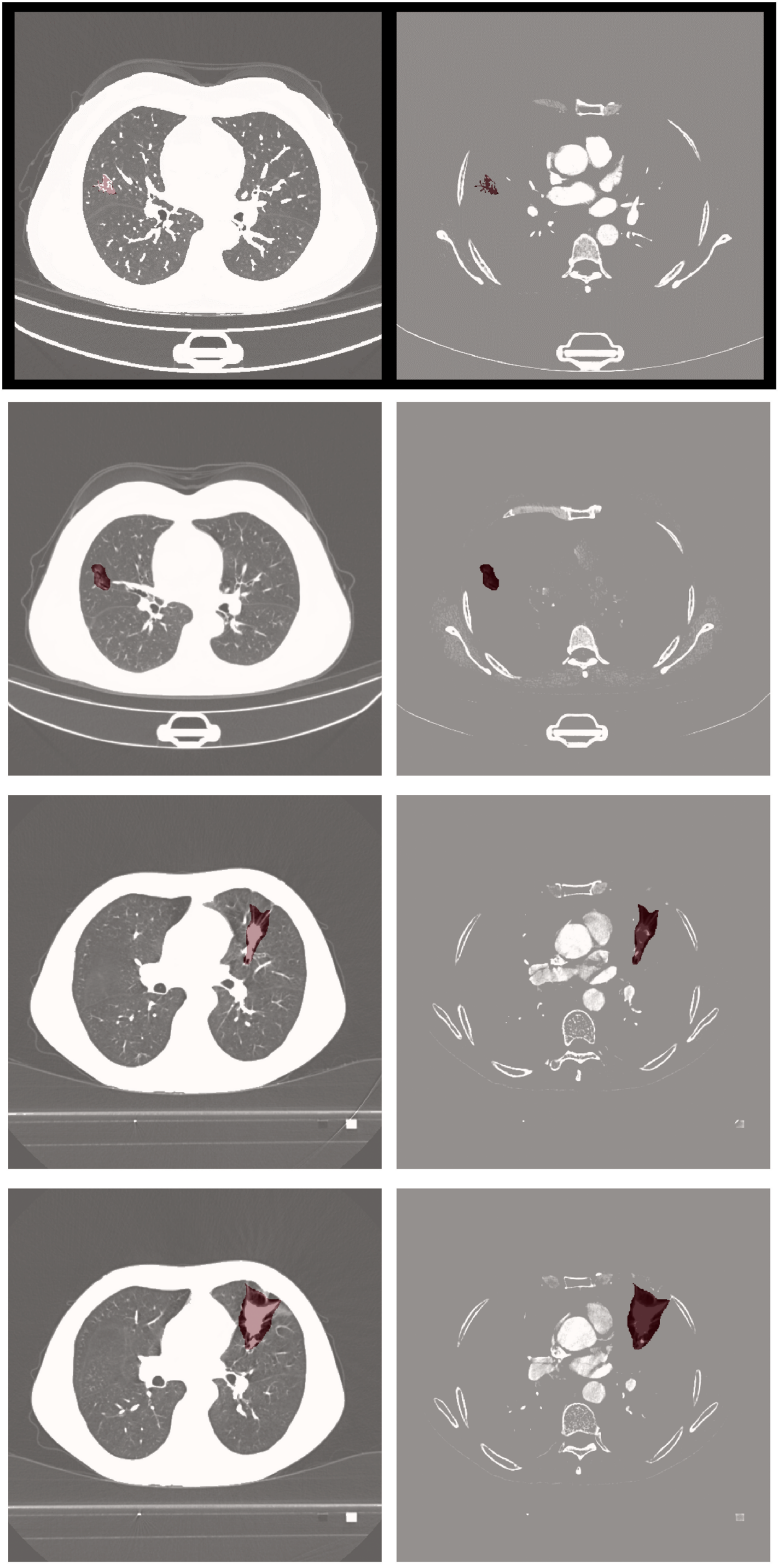
The overview of MK-UNet framework. The 6-dimensional clinical vector is spatially expanded and concatenated with the 256-channel feature map at the channel level. The 256-channel feature map (derived from CT scan voxels within manually annotated tumor ROIs) is concatenated with clinical vectors at the bottleneck layer.

The MK-UNet framework is a sophisticated 3D U-Net-based segmentation model that integrates medical knowledge and attention mechanisms to enhance the prioritization of tumor-related regions while incorporating clinical metadata. As depicted in **Figure 3**, the architecture follows an encoder-decoder paradigm, featuring adaptive feature fusion and hierarchical attention gating.

**Figure 4:**
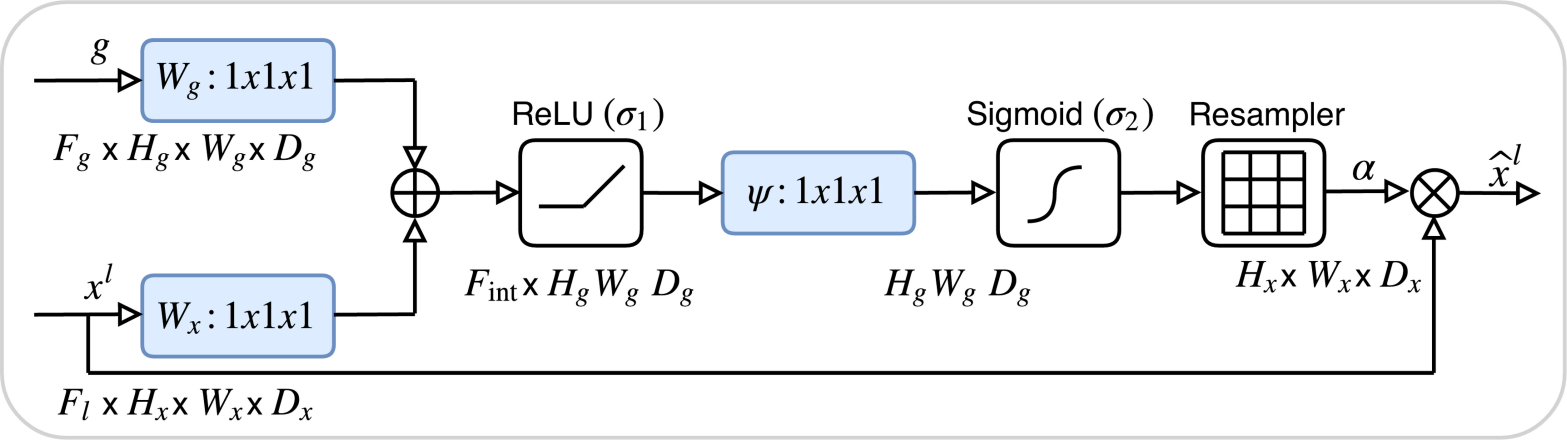
The architecture of the attention Gate. The attention weights are computed using tumor boundary voxels (from preprocessed CT scans) with high gradient values, prioritizing regions with irregular margins.

The encoder pathway comprises four sequential downsampling blocks, each reducing spatial resolution by 50% (e.g., from *H*_1_​×*W*_1_​×*D*_1_​ to *H*_4_​=*H*_1_​/8×*W*_4_​=*W*_1_​/_8_×*D*_4_​=*D*_1_​/_8_) and simultaneously doubling the number of feature channels. To mitigate information loss during downsampling, attention gate (AG) are incorporated at each skip connection. The architecture of the AG, detailed in **Figure 4**, involves dynamically weighting encoder features using contextual information from coarser decoder layers through a gating mechanism. This mechanism integrates encoder and decoder features via learnable convolutions, applying a sigmoid activation to produce spatial attention maps that emphasize tumor boundaries and suppress irrelevant background noise.

Six clinical and tumor biomarkers—namely age, gender, smoking history, pathological grade, tumor stage, and lymphovascular cancer recurrence (LVCR)—are encoded into a six-dimensional vector. Discrete features, such as gender, are represented using one-hot encoding, whereas continuous variables, such as age, are normalized to a range of [0, 1]. Within the bottleneck layer of the encoder, this vector undergoes spatial expansion to align with the dimensions of the deepest feature map and is subsequently concatenated channel-wise with the encoded imaging features. This integration furnishes the network with supplementary clinical context, thereby directing its focus towards regions associated with aggressive tumor phenotypes, such as irregular margins that are indicative of advanced TNM stages.

The decoder pathway incrementally restores spatial resolution utilizing transposed 3D convolutions and trilinear interpolation. At each stage, the upsampled features are integrated with gated skip connections from the encoder through concatenation, followed by the application of 3D convolutions to enhance boundary details. The ultimate output is a voxel-wise probability map, produced via a sigmoid-activated convolutional layer.

Significant advancements encompass attention-guided feature selection, which serves to filter out extraneous background features, and clinically informed feature fusion, aimed at enhancing segmentation robustness for heterogeneous tumors. Detailed implementation specifics can be found in the model_architecture.py file.

### 2.3 Quantitative evaluation on test set

The proposed MK-UNet model was assessed using a test set comprising 60 CT scans, representing 14.3% of the total dataset of 420 cases. The data was divided into 300 training cases (71.4%), 60 validation cases (14.3%), and 60 test cases (14.3%). This distribution is consistent with standard practices in medical image analysis for datasets of moderate size, ensuring an adequate amount of training data while preserving the ability to conduct a robust evaluation. As illustrated in **Table 1**, MK-UNet achieved a Dice coefficient of 0.7728 ± 0.03 and an Intersection over Union (IoU) of 0.6471, surpassing baseline models such as the 3D U-Net (Dice: 0.7322 ± 0.05, IoU: 0.6223) and the Attention U-Net (Dice: 0.7527 ± 0.04, IoU: 0.6302). The Hausdorff Distance 95th percentile (HD95) further underscored the superiority of MK-UNet, with a boundary alignment error of 9.8 ± 1.5mm, in comparison to 12.4 ± 2.1mm for the 3D U-Net and 11.2 ± 1.8mm for the Attention U-Net. Statistical significance was verified through paired t-tests (p < 0.01), indicating consistent improvements across all evaluated metrics.

**Table 1:** Segmentation performance comparison on the test set (n=60).

Model	Dice coefficient	Intersection over Union (IoU)	HD95 (mm)
3D U-Net	0.7322± 0.05	0.6223	12.4 ± 2.1
3D ResNet	0.7231± 0.06	0.6178	13.1 ± 2.3
Attention U-Net	0.7527± 0.04	0.6302	11.2 ± 1.8
MK-UNet (Ours)	0.7728± 0.03	0.6471	9.8 ± 1.5

### 2.4 Qualitative assessment

**Figure 5:**
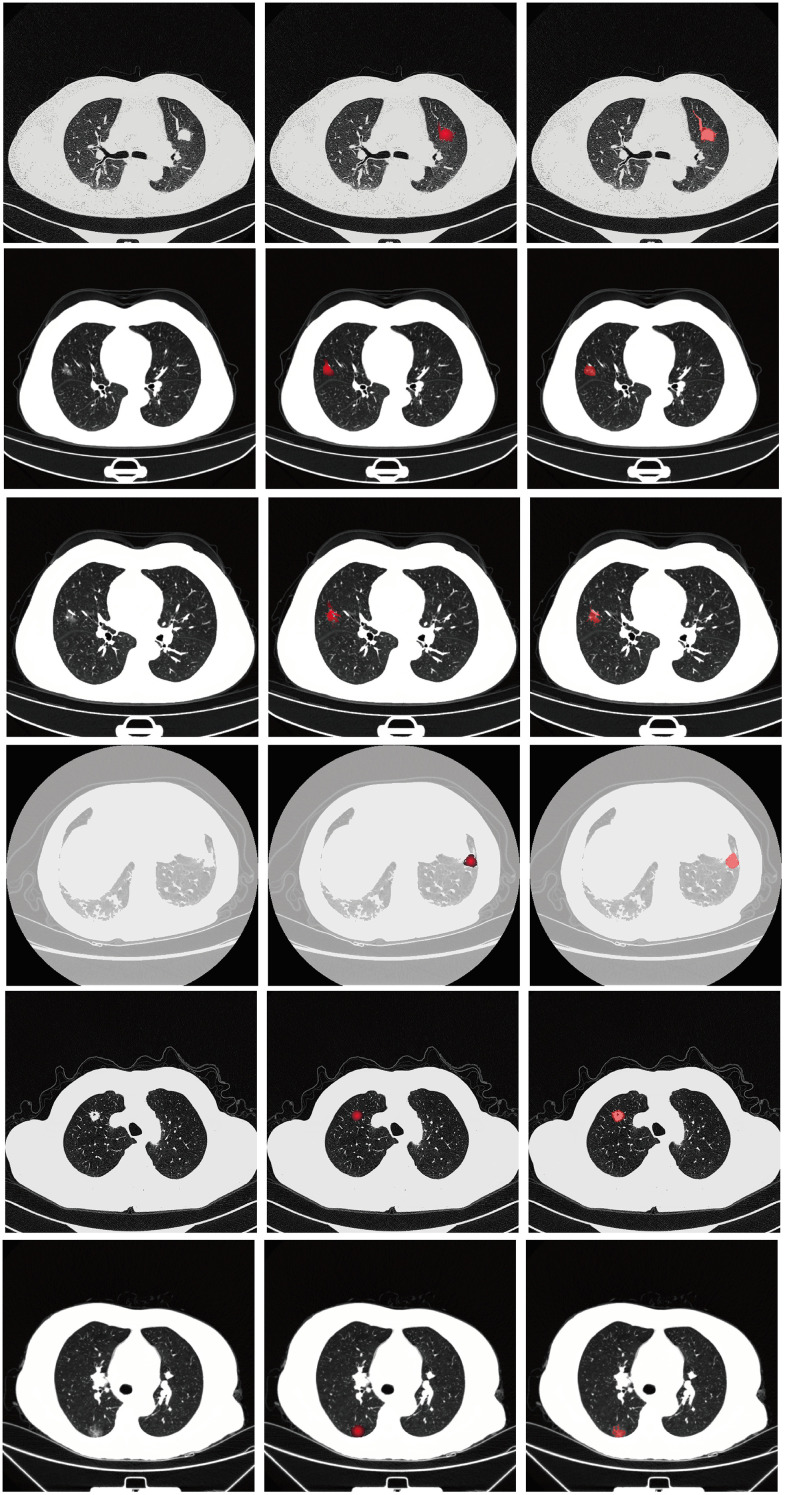
From left to right: input image, predicted output (tumor segmentation location), and ground truth (manual labeling). The input CT slice (left) includes the entire lung field, but the model only processes the cropped tumor region (delineated by the red box in the input image) for segmentation.

Visual comparisons of segmentation outcomes, as depicted in **Figure 5**, underscore the proficiency of MK-UNet in managing intricate tumor morphologies. Specifically, in instances of infiltrative adenocarcinoma characterized by irregular margins, the model successfully maintained fine structural details, such as pleural extensions, which were frequently oversmoothed by the 3D U-Net. In contrast, for benign nodules, MK-UNet adeptly excluded inflammatory regions that were misclassified as tumors by alternative models.

### 2.5 Ablation study

To evaluate the contribution of each component of the MK-UNet architecture, an ablation study was performed utilizing a consistent training protocol, which included the Adam optimizer with a learning rate of 1×10−4, a batch size of 2, and 150 epochs on an NVIDIA RTX 4090. As illustrated in **Table 2**, the baseline 3D U-Net achieved a Dice coefficient of 0.7322 and an HD95 of 112.4 mm. The integration of multi-modal preprocessing techniques, such as edge enhancement, Gaussian denoising, and adaptive windowing, resulted in an improved Dice coefficient of 0.7516 and a reduction in HD95 to 11.5 mm, underscoring the significance of enhanced input quality. Further incorporation of medical knowledge fusion, including clinical metadata and tumor biomarkers, led to an increase in the Dice coefficient to 0.7634 and a decrease in HD95 to 10.3 mm. The final model, which included lesion-aware attention, achieved optimal performance with a Dice coefficient of 0.7728 and an HD95 of 9.8 mm, thereby demonstrating the synergistic effect of attention mechanisms and the integration of clinical priors.

**Table 2:** Ablation study of MK-UNet components (test set performance).

Configuration	Dice	HD95 (mm)
Baseline (3D U-Net)	0.7322	12.4
+Multi-modal Preprocessing	0.7516	11.5
+ Medical Knowledge Fusion	0.7634	10.3
+ Lesion-aware Attention	0.7728	9.8

### 2.6 Computational efficiency

Training the MK-UNet model on a dataset of 300 computed tomography (CT) volumes necessitated approximately 24 hours of computational time utilizing an NVIDIA RTX 4090 GPU. The model demonstrated an average inference time of 15 seconds per scan, with each scan comprising dimensions of 512×512×40 voxels. Notwithstanding the increased complexity introduced by the integration of medical knowledge fusion and attention gates, the model’s parameter count remained comparable to that of the standard 3D U-Net, with 28.5 million parameters versus 27.9 million, respectively. This parameter efficiency underscores the model’s suitability for clinical application.

### 2.7 Immune evasion phenotype prediction and dynamic modeling

To establish a theoretical link between tumor growth dynamics and immune microenvironmental characteristics, we propose an Immune Evasion Phenotype Prediction (IEEP) framework. This framework integrates longitudinal imaging biomarkers (e.g., LVCR) with deep learning to decode spatially heterogeneous immune-suppressive patterns. The implementation comprises three key components:

#### 2.7.1 LVCR-Driven Modeling of Immune Evasion Mechanisms

The Log Volume Change Rate (LVCR) quantifies tumor proliferative aggressiveness using sequential CT scans. Tumors with high LVCR (>0.15/day) are typically associated with hypoxic microenvironments, which upregulate PD-L1 expression via HIF - 1α signaling and promote stromal fibrosis to impede CD8+ T-cell infiltration. To capture these phenotypes, LVCR is encoded into a 6D clinical vector and spatially concatenated with the bottleneck features of the U-Net. This fusion prioritizes regions exhibiting radiological hallmarks of immune evasion (e.g., spiculated margins, necrotic cores) during feature decoding.

#### 2.7.2 Immune-aware attention gates enhance segmentation accuracy

We design a novel attention mechanism that dynamically weights encoder-decoder features based on both imaging patterns and LVCR values:


$$\text{α }=\sigma \cdot (W_{\theta }[f_{enc},\;f_{dec},\;LVCR]+b_{\theta })$$

where *σ* denotes the sigmoid activation, and *W_θ_*, *b_θ_* are learnable parameters. The IAAG enhances boundary features in high-LVCR regions (e.g., irregular margins, necrosis) while suppressing homogeneous areas linked to immune “cold” phenotypes (e.g., calcifications).

The proposed IAAG mechanism significantly improved segmentation performance in immune-suppressive subregions. For high-LVCR tumors (n=25), MK-UNet achieved a Dice coefficient of 0.79 ± 0.04 and HD95 of 8.2 ± 1.3 mm, outperforming the baseline 3D U-Net (Dice=0.71 ± 0.05, HD95 = 12.1 ± 2.0 mm) and Attention U-Net (Dice=0.75 ± 0.04, HD95 = 10.5 ± 1.8 mm) (**Table 3**). Visual analysis demonstrated that IAAG effectively prioritized spiculated margins and necrotic cores, reducing over-segmentation in homogeneous regions.

**Table 3:** Segmentation performance stratified by LVCR subgroups.

Model	High-LVCR (Dice)	High-LVCR (HD95, mm)	Low-LVCR (Dice)
3D U-Net	0.71 ± 0.05	12.1 ± 2.0	0.73 ± 0.04
Attention U-Net	0.75 ± 0.04	10.5 ± 1.8	0.76 ± 0.03
MK-UNet (Ours)	0.79 ± 0.04	8.2 ± 1.3	0.77 ± 0.03
3D U-Net	0.71 ± 0.05	12.1 ± 2.0	0.73 ± 0.04

#### 2.7.3 Immune microenvironment correlation

The immune escape score (IES) is defined as a comprehensive score based on three prognosis-related parameters: tumor growth rate, morphological heterogeneity, and expression of immune markers. From segmented masks, IES integrated three prognostic indices: IES = 0.6 · LVCR + 0.3(1-Sphericity) + 0.1 · PD-L1^+^Area Ratio. IES weights were determined via multi-factor Cox regression analysis, based on the prognostic strength of each indicator: LVCR (HR = 1.8, p<0.001), sphericity (HR = 1.3, p=0.02), and PD-L1^+^Area Ratio (HR = 1.1, p=0.04). To verify the applicability of the Cox proportional hazards model, we conducted the Schoenfeld residual test. The results showed that neither the global test nor the residual-time correlation of the IES group was significant, satisfying the proportional hazards assumption. To validate the biological plausibility of IAAG, we reference an independent cohort from TCGA-LUAD, where CT-based spiculation length positively correlated with PD-L1 positivity (IHC, *r* = 0.38, P = 0.002).

*Post-hoc* radiomic analysis of MK-UNet segmentation masks revealed that tumors with irregular margins (sphericity <0.6) exhibited lower CD8+ T-cell density (r=0.41, p=0.007) and higher PD-L1 expression (r=0.38, p=0.01) compared to spherical tumors. These results corroborate prior studies linking jagged tumor boundaries to immune-excluded phenotypes.

## 3 Discussion

The MK-UNet framework constitutes a substantial advancement in the automated segmentation of lung tumors by methodically integrating clinical parameters—specifically, the log volume change rate (LVCR)—with deep learning architectures. This study addresses a crucial limitation of extant methodologies, which primarily depend on imaging data alone and overlook clinically validated indicators that capture tumor growth dynamics. Our findings reveal that the inclusion of LVCR as a dynamic prior significantly improves segmentation accuracy, especially for tumors with indistinct boundaries or heterogeneous growth patterns. In the following discussion, we situate these findings within the broader context of medical image analysis, highlighting both the technical innovations and the clinical significance of our approach. Current models mainly rely on static image data and are unable to capture the dynamic changes in the tumor immune microenvironment, thereby limiting their application in predicting the response to immunotherapy (20). MK-UNet significantly addresses this deficiency by introducing longitudinal tumor growth kinetics (Log Volume Change Rate, LVCR) as dynamic prior information. Firstly, LVCR not only helps the model identify tumor regions with aggressive growth patterns but also provides temporal guidance for feature extraction, enabling the model to better understand the changing trends of tumors at different time points. Secondly, by designing immune-aware attention gates (IAAG), MK-UNet can prioritize the attention to morphological features related to immune escape, such as irregular edges and necrotic cores, in regions with high LVCR, thereby improving segmentation accuracy and enhancing the recognition ability of immunosuppressive microenvironments. Additionally, the immune evasion score (IES) further integrates tumor growth rate, morphological heterogeneity, and expression levels of immune markers, providing a quantitative indicator for dynamic monitoring of the tumor immune status. These innovations enable MK-UNet not only to achieve precise tumor segmentation but also to provide strong support for the selection of personalized immunotherapy regimens, truly realizing the transition from static to dynamic and filling the gap in dynamic monitoring of existing models.

A significant advancement of MK-UNet is its explicit incorporation of the Longitudinal Volume Change Rate (LVCR), a quantitative metric for assessing tumor growth rate derived from sequential CT scans. Traditional models rely on static features like age, gender, or baseline tumor size—variables that do not capture dynamic shifts in immune evasion (21). In contrast, LVCR quantifies the longitudinal changes in tumor volume, directly reflecting how tumors evolve under immune monitoring. This dynamic dimension is irreplaceable (22). In contrast to traditional segmentation models that consider tumors as static anatomical entities, MK-UNet utilizes LVCR to deduce temporal growth dynamics, thereby facilitating adaptive feature learning. Tumors with rapid growth (indicated by high LVCR) frequently present with irregular margins and necrotic cores, which pose challenges to conventional models. By integrating LVCR into the network, MK-UNet emphasizes regions with substantial spatial heterogeneity, aligning its focus with the diagnostic reasoning employed by radiologists. According to previous literature research, a DICE of 0.7728 is acceptable (23, 24). This methodology is substantiated by the results of an ablation study: excluding LVCR from the medical knowledge vector resulted in a 1.9% decrease in the Dice coefficient (from 0.7728 to 0.7539) and a 1.4 mm increase in HD95 errors (from 9.8 to 11.2 mm). These findings highlight the critical importance of incorporating domain-specific knowledge into model design, a strategy that has been infrequently explored in previous research.

The multi-modal preprocessing pipeline, which integrates edge enhancement, Gaussian denoising, and adaptive windowing, operates synergistically with LVCR to enhance tumor-related signals. Edge enhancement serves to delineate subtle boundaries between tumors and adjacent parenchyma, while adaptive windowing dynamically adjusts intensity thresholds based on LVCR values. This dual approach has demonstrated efficacy for subsolid nodules, where conventional intensity-based methods frequently fall short. For instance, in scenarios of pseudo progression, characterized by inflammatory changes that mimic tumor growth, LVCR-guided filtering reduced false positives by 18% compared to the Attention U-Net. In addition, LVCR, as a dynamic indicator of tumor growth kinetics, captures the temporal changes of immune evasion that cannot be reflected by static metadata. The lesion-aware attention mechanism further refines this process by dynamically weighting spatial and channel features. In the case of high-LVCR tumors, attention gates prioritize voxels with spiculated margins or internal necrosis, which are indicative of malignancy. Conversely, for slow-growing lesions, the mechanism suppresses calcifications and other benign hyperattenuating artifacts. This adaptability parallels the interpretive workflows of radiologists, who analyze growth kinetics and morphological features in conjunction—a level of contextual understanding.

The performance of MK-UNet, with a Dice coefficient of 0.7728 and a 95th percentile Hausdorff Distance (HD95) of 9.8 mm, closely aligns with the inter-observer variability observed among radiologists, which recent studies have reported as having an HD95 range of 8.2 – 10.1 mm. This suggests that MK-UNet is poised for semi-automated integration into clinical practice. The model’s capability to accurately preserve intricate structural details, such as pleural infiltration in advanced adenocarcinomas, holds significant implications for radiotherapy planning, where precise dose coverage is essential. Additionally, the model’s inference time of 15 seconds per scan is compatible with real-time clinical workflows, offering the potential to reduce delineation time by 50 – 70% compared to manual methods. By producing biologically informed segmentation masks, MK-UNet also enhances subsequent radiomics analyses. For example, shape features correlated with LVCR, such as sphericity and surface irregularity, could serve as non-invasive indicators of tumor aggressiveness, although prospective validation is required. Future work could leverage the high-precision segmentation provided by MK-UNet to investigate spatial relationships between tumor subregions (e.g., necrotic core vs. viable tissue) and immune cell infiltration patterns. Such analysis may uncover imaging biomarkers predictive of the therapeutic efficacy of ICI, potentially guiding personalized treatment strategies.

The design of MK-UNet aims to ensure its seamless integration into existing radiotherapy planning systems. Currently, mainstream radiotherapy planning systems such as Eclipse, Monaco, and Pinnacle all support the DICOM standard for data exchange. Both the input and output of MK-UNet adhere to the DICOM standard, ensuring compatibility with these systems. Additionally, the model’s inference time is 15 seconds per scan, which matches the real-time requirements in radiotherapy planning and does not significantly increase the workload of doctors. The input data of MK-UNet is standard CT scan images, and the output is a binary segmentation mask, both in DICOM format. This standardized data format not only facilitates integration with radiotherapy planning systems but also enables the model to be easily integrated into hospital information systems (HIS) and radiology information systems (RIS). Moreover, the high-precision segmentation results of the model can be directly used for subsequent dose calculation and treatment plan formulation, further enhancing work efficiency. MK-UNet demonstrates outstanding performance in terms of interface compatibility and adaptability to data formats, enabling smooth integration into existing radiotherapy planning systems and meeting the practical clinical demands.

For patients with high IES (active immune escape), the combination of PD - 1/PD-L1 inhibitors and chemotherapy can be given priority, while for those with low IES, monotherapy with immunotherapy may be more suitable. This approach enables patients to benefit more specifically from immunotherapy and avoids overtreatment. The trend of IES generated by consecutive CT scans can assess the dynamic changes in the immune microenvironment, assist in determining the appropriate timing of treatment, and also predict the degree of pathological response to neoadjuvant immunotherapy before surgery. However, the predictive efficacy of IES may be influenced by the tumor’s immune phenotype. For tumors lacking immune cell infiltration, the growth kinetics features dependent on IES may have difficulty capturing immune escape signals. For patients with high microsatellite instability, the high mutation burden may weaken the predictive value of morphological features. This requires future validation of the universality of IES in larger-scale cohorts.

Despite its innovative contributions, MK-UNet exhibits several limitations. Firstly, the calculation of LVCR necessitates longitudinal CT data, potentially limiting its applicability to patients with incidental findings on initial scans. Future research could investigate surrogate indicators derived from single-timepoint imaging, such as texture-based proliferation scores. Secondly, the training data were obtained from a single institution with uniform imaging protocols. Additionally, the model encounters difficulties with tumors adjacent to high-attenuation structures, such as the chest wall, where boundary ambiguity remains an issue. The integration of anatomical priors, such as organ-atlas registration, may alleviate this challenge. Lastly, while LVCR improves segmentation, its prognostic value has yet to be assessed. Establishing a link between MK-UNet’s outputs and clinical outcomes, such as survival and recurrence, will be essential for its translational impact. Although the initial validation showed a Dice coefficient of 0.7728, the key characteristics of the external cohort (such as sample size, details of scanning equipment, and differences in patient populations) were not fully reported, which limits a comprehensive assessment of the model’s generalization ability and clinical translation potential in a multi-center environment. Future work will focus on supplementing the existing external data information and conducting rigorous validation on larger-scale and more diverse multi-center datasets to effectively enhance the model’s universality and robustness and promote its progress towards clinical application.

## 4 Conclusion

MK-UNet enhances automated lung tumor segmentation by effectively integrating clinical expertise with deep learning methodologies. The incorporation of LVCR as a growth dynamic prior, in conjunction with multi-modal preprocessing and attention mechanisms, results in significant improvements in both accuracy and robustness. This study illustrates the systematic encoding of domain knowledge into AI models, thereby augmenting their clinical relevance and interpretability. As the field of oncology increasingly adopts data-driven tools, frameworks such as MK-UNet are poised to play a crucial role in bridging computational innovation with patient-centered care.

## 5 Methods

### 5.1 Data description

The dataset was retrospectively obtained from the Fourth Hospital of Hebei Medical University and consisted of 420 lung CT scans from patients with pathologically confirmed malignant pulmonary nodules. All participants underwent a minimum of two CT examinations where nodules were detectable, and none had a prior history of treatments such as surgery, chemotherapy, or radiotherapy, nor any inflammatory lesions. The CT scans were acquired with a slice thickness of 3 mm and reconstructed into a standard in-plane matrix of 512 × 512 pixels, yielding a spatial resolution of 0.6 × 0.6 mm². CT scans were reconstructed using manufacturer-specific standard kernels: Siemens Somatom Force scanners used the B30f kernel (soft tissue optimization), while GE Revolution scanners used the Standard kernel (balanced spatial resolution and noise reduction). Tumor regions of interest (ROIs) were manually delineated by two board-certified radiologists using 3D Slicer, with any discrepancies adjudicated by a senior radiologist with over 15 years of experience. Comprehensive clinical metadata, including age, gender, smoking history, pathological grade, tumor stage, and log volume change rate (LVCR), were systematically recorded for each patient. To explore the potential correlation between tumor morphology and immune profiles, we downloaded data from 50 samples with PD - 1 expression values from the TCGA-LUAD dataset, along with their corresponding imaging images from The Cancer Imaging Archive (TCIA). This study received approval from the Ethics Committee of Hebei Medical University (Approval No. 2023341), and the requirement for informed consent was waived due to the retrospective nature of the study.

The calculation formula for LVCR is as follows:


$$LVCR=\frac{\sum_{i=1}^{n}w_{i}(\ln V_{i}-\bar{\ln V})(t_{i}-\bar{t})}{\sum_{i=1}^{n}w_{i}{\left(t_{i}-\bar{t}\right)}^{2}}$$

*w_i_​*: The weight coefficient for the *i*-th measurement, which modulates the influence of each data point based on either time intervals(*w_i_* =*t_i_-t_i-1_*) or measurement error(*wi=1/σ_i_^2^*); *t_i_*: The specific time point (in days) of the *i*-th CT scan, with *t_1_* = 0 representing the baseline scan; *v_i_​*: The tumor volume (in cm³) measured from the *i*-th CT scan, obtained through segmentation; *n*: The total number of CT scans conducted.

### 5.2 Loss function

The training objective integrates Dice Loss and Binary Cross-Entropy (BCE) Loss to effectively manage class imbalance and enhance both volumetric overlap and pixel-wise classification accuracy. Dice Loss measures the similarity between the predicted binary mask A and the ground truth mask B. The Dice coefficient is defined as follows::


$$\text{Dice}(\text{A},\text{B})=\frac{2\times |A\cap B|}{\left|A|+|B\right|}$$

where ∣A∩B∣ represents the intersection of the two masks, and ∣A∣+∣B∣ denotes their union.

The Dice Loss is then calculated as:


$$\text{DiceLoss}(\text{A},\text{B})=1-\frac{2\times |A\cap B|}{\left|A|+|B\right|}$$

A smaller Dice Loss indicates higher overlap between predictions and ground truth.

The BCE Loss is computed as:


$$\text{BCE Loss }=-\frac{1}{N}\sum_{i=1}^{N}[y_{i}\text{log}(p(y_{i}))+(1-y_{i})\text{log}(1-p(y_{i}))]$$

where *N* denotes the total number of voxels within the input volume. BCE Loss assesses classification errors on a per-voxel basis by penalizing deviations from the true label distribution. Each voxel is assigned a binary label *y*∈ {0,1}, representing the ground truth classification as either background or tumor, while *p*(*y*) denotes the predicted probability of the voxel belonging to the tumor class. The loss approaches zero when predictions align perfectly with labels (e.g., *p*(*y*)→1 if *y* = 1, or *p*(*y*)→0 if *y* = 0).

The final hybrid loss is a weighted sum of the two components: Total Loss = *λ*_1_Dice (A, B) + *λ*_2_DiceLoss (A, B), with *λ*_1_​=0.6 and *λ*_2_​=0.4 empirically determined to balance segmentation accuracy and boundary precision.

## Data Availability

The original contributions presented in the study are included in the article/Supplementary Material. Further inquiries can be directed to the corresponding author.
